# Determination of Optimal Antigen Yield and Virus Inactivation Conditions for the Production of the Candidate Foot-and-Mouth Disease Recombinant Vaccine Strain Asia1 Shamir-R in a Bioreactor

**DOI:** 10.3390/v16030457

**Published:** 2024-03-16

**Authors:** Jae Young Kim, Sun Young Park, Gyeongmin Lee, Sang Hyun Park, Jong-Sook Jin, Dohyun Kim, Jong-Hyeon Park, Seong-Yun Jeong, Young-Joon Ko

**Affiliations:** 1 Animal and Plant Quarantine Agency, Gimcheon-si 39660, Republic of Korea; ivorikim@korea.kr (J.Y.K.); lgm6004@korea.kr (G.L.); doh936@gmail.com (D.K.);; 2 Department of Biomedical Science, Graduate School, Catholic University of Daegu, Daegu 38430, Republic of Korea; jsymicro@cu.ac.kr

**Keywords:** foot-and-mouth disease, type Asia1, vaccine, antigen, bioreactor

## Abstract

Since the foot-and-mouth disease (FMD) outbreak in South Korea in 2010–2011, vaccination policies utilizing inactivated FMD vaccines composed of types O and A have been implemented nationwide. However, because type Asia1 occurred in North Korea in 2007 and intermittently in neighboring countries, the risk of type Asia1 introduction cannot be ruled out. This study evaluated the antigen yield and viral inactivation kinetics of the recombinant Asia1 Shamir vaccine strain (Asia1 Shamir-R). When Asia1 Shamir-R was proliferated in shaking flasks (1 L), a 2 L bioreactor (1 L), and a wave bioreactor (25 L), the antigen yields were 7.5 μg/mL, 5.2 μg/mL, and 3.8 μg/mL, respectively. The optimal FMDV inactivation conditions were 2 mM BEI at 26 °C and 1.0 mM BEI at 37 °C. There was no antigen loss due to BEI treatment, and only a decrease in antigen levels was observed during storage. The sera from pigs immunized with antigen derived from a bioreactor exhibited a neutralizing antibody titer of approximately 1/1000 against Asia1 Shamir and Asia1/MOG/05 viruses; therefore, Asia1 Shamir-R is expected to provide sufficient protection against both viruses. If an FMD vaccine production facility is established, this Asia1 Shamir-R can be employed for domestic antigen banks in South Korea.

## 1. Introduction

Foot-and-mouth disease (FMD), which affects cloven-hoofed livestock, is economically important because of its highly contagious nature [1]. FMD virus (FMDV), the causative agent of FMD, belongs to the *Aphthovirus* genus of the *Picornaviridae* family [2]. FMDV has a positive-sense, single-stranded RNA genome that is translated into a polyprotein, which is further cleaved into structural and nonstructural proteins [1,3,4]. Among the seven serotypes (O, A, Asia1, C, and SAT 1–3) of FMD, three (O, A, and Asia1) have been reported in Asian countries. The serotype Asia1 is grouped into nine genetic groups (G-I to G-IX) based on nucleotide variations in the VP1 sequence [5]. 

The capsid of FMDV consists of four proteins called VP1, VP2, VP3, and VP4. Initially, VP0, a combination of VP4 and VP2, assembles into protomers with VP3 and VP1, five protomers assembles into the pentamer of 12S, and twelve pentamers come together to assemble the viral precursor of 75S. Upon entry of the RNA into the capsid, VP0 is cleaved into VP2 and VP4, leading to an intact FMDV (146S). When subjected to sucrose density gradient ultracentrifugation, the intact 146S, 75S, and 12S are separated in the sucrose density gradient tube. Normally, FMDV is subject to degradation to 12S by heat or pH. The capsid components of the 146S and 12S are the same, but there is a huge difference in immunogenicity between the 146S and 12S forms. According to a previous report [6], the 146S is more immunogenic by 100 times against FMDV than the 12S. Therefore, the antigen yield of an FMD vaccine plant is directly related to the economics of the plant, leading to the competitiveness of FMD vaccine.

Since the massive FMD outbreak in South Korea in 2010–2011, vaccination policies have been implemented nationwide with bivalent FMD vaccines comprising types O and A. However, since type Asia1 occurred in North Korea in 2007 and intermittently in neighboring countries [7], the risk of type Asia1 introduction cannot be ruled out. Therefore, Korea has stockpiled type Asia1 vaccines in the form of an antigen bank. Meanwhile, the current FMD vaccines are entirely imported from abroad. In case an FMD vaccine manufacturing facility is built in Korea in the future, our institute has been developing various types of FMD vaccine seed viruses. 

Since the Asia1 G-VIII that was reported in 2017 in Asia, was found to match with the Asia1 Shamir vaccine, which is known to be the only international vaccine strain of Asia1, the recombinant Asia1 Shamir vaccins strain (Asia1 Shamir-R) was constructed and shown to provide protection against Asia1 Shamir [8]. This study aimed to determine whether Asia1 Shamir-R can be commercially utilized for the FMD vaccine. In other words, we investigated whether the antigen yield of Asia1 Shamir-R is sufficiently high when scaled up from a flask to a bioreactor. Also, since the FMD vaccine is an inactivated vaccine, we wanted to ensure that Asia1 Shamir-R was completely inactivated by BEI treatment. Furthermore, it was necessary to ensure if the inactivated Asia1 Shamir-R antigen derived from a bioreactor was still immunogenic in pigs.

## 2. Materials and Methods

### 2.1. Cells and Viruses

BHK-21 suspension cells were established in serum-free media, as previously described [9]. They were adapted for growth in a ProVero-1 cell culture medium (Lonza Ltd., Basel, Switzerland) via incubation at 110 rpm in a shaking incubator at 37 °C with 5% CO_2_. Porcine kidney (LFBK) cells (Plum Island Animal Disease Center, Orient, NY, USA) and BHK-21 adherent cells were cultured in Dulbecco’s modified Eagle medium (DMEM; Thermo Fisher Scientific, Waltham, MA, USA). Asia1 Shamir-R used in this study was derived from an infectious Asia1 Shamir virus clone (GenBank accession No. JF739177.1), as previously reported [7]. The full genome cDNA of the Asia1 Shamir virus was cloned under the T7 RNA polymerase of the pBluescript SK II vector (Stratagene, San Diego, CA, USA). The viruses used for the virus neutralization test were Asia 1 Samir and Asia1 MOG/05-R [10].

### 2.2. Determination of Optimal Conditions for FMDV Proliferation

The BHK-21 suspension cells were cultured in Cellvento BHK-200 medium (Merck, Darmstadt, Germany) starting with 3 × 10^5^ cells/mL in the volume of 100 mL until they reached a density of around 3 × 10^6^ cells/mL for 3.5 days. Then, the Asia1 Shamir-R was inoculated onto the cells at a multiplicity of infection (MOI) of 0.001, 0.005, 0.01, and 0.05 in a shaking incubator at 37 °C with 5% CO_2_. Viruses were harvested at 12, 16, 20, and 24 h post infection (hpi) and clarified by centrifugation at 3000× *g* for 20 min at 4 °C to remove cell debris. 

### 2.3. Virus Titration

Virus titers were determined in adherent BHK-21 cells via endpoint titration using the Spearman–Kärber calculation and presented as the tissue culture infectious dose affecting 50% of the cultures (TCID_50_) per mL [11]. 

### 2.4. Quantification of FMDV Particles

The quantity of FMDV particles was measured using a previously described method [12]. Briefly, the viral infection supernatant was treated with chloroform (Merck KGaA, Darmstadt, Germany) at a ratio of 1:1 (*v*/*v*) and mixed via vigorous inversion for 5 min. The mixture was centrifuged at 3000× *g* for 15 min at 4 °C, and then, the aqueous phase on top of the organic solvent was collected. The samples were treated with benzonase (Sigma-Aldrich, St. Louis, MO, USA) at a final concentration of 0.025 units/μL and incubated at 37 °C for 1 h with shaking. After digestion, the samples were centrifuged at 16,000× *g* for 10 min at 4 °C to obtain a clear sample. The FMDV intact particles in the samples were measured by loading the samples onto a high-performance liquid chromatography (Agilent Technologies, Santa Clara, CA, USA) fitted with a TSKgel G4000PWXL column (TOSOH Bioscience, Tokyo, Japan). Finally, the peak area was integrated for the quantification of FMDV particles.

### 2.5. Preparation of the Antigen Using a Shaking Flask, a 2 L Bioreactor, and a 25 L Wave Bioreactor

For flask culture, when the BHK-21 suspension cells with 3 × 10^5^ cells/mL in the volume of 28 mL, 140 mL, and 700 mL were grown for 3.5 d up to approximately 3 × 10^6^ cells/mL in Cellvento BHK-200 medium, the Asia1 Shamir-R was inoculated onto the cells at 0.0005 MOI with 12 mL, 60 mL, and 300 mL of fresh Cellvento BHK-200 medium in a shaking incubator at 37 °C with 5% CO_2_ for 24 h. For the 2 L bioreactor (Sartorius, Goettingen, Germany), the 700 mL of Cellvento BHK-200 cell culture media was transferred to a 2 L bioreactor with an initial cell density of 3 × 10^5^ cells/mL. The bioreactor was equipped with probes to measure and control the temperature and dissolved oxygen at 37 °C and 45% air saturation. The pH was controlled in the range of 7.2–7.4 by adding CO_2_ and 0.5 M NaOH solution. Agitation speed was maintained at 150 rpm. After the cell density reached approximately 3 × 10^6^ cells/mL, 300 mL of the Cellvento BHK-200 cell culture medium was added to the bioreactor. Subsequently, Asia1 Shamir-R was added to fresh media in the bioreactor at 0.0005 MOI. The FMDV-infected supernatants were collected at 24 hpi. For the 25 L Biostat RM rocker with Flexsafe RM bags (Sartorius, Goettingen, Germany), the 17.5 L of Cellvento BHK-200 cell culture media was transferred to the RM bag with an initial cell density of 3 × 10^5^ cells/mL. The RM bag was equipped with probes to measure and control the temperature and dissolved oxygen at 37 °C and 50% air saturation. The pH was controlled in the range of 7.2–7.4 by adding CO_2_ and 1 M NaOH solution. The rocking speed was maintained at 100 rpm. After the cell density reached approximately 3 × 10^6^ cells/mL, 7.5 L of Cellvento BHK-200 cell culture medium was added to the RM bag. Subsequently, Asia1 Shamir-R was added to fresh media in the bioreactor at 0.0005 MOI. The FMDV-infected supernatants were collected at 24 hpi. 

### 2.6. Inactivation Kinetics of FMDV

Binary ethylenimine (BEI) was prepared by dissolving bromoethylamine hydrobromide (Sigma-Aldrich) in 10 mL of 0.2 N sodium hydroxide solution (Sigma-Aldrich) to a concentration of 0.1 M. The solution was then incubated at 37 °C in a shaking incubator at 100 rpm for 1 h. The pH of the solution was adjusted to a range of 8.5–9. The solution was prepared before use. The in-process quality control of the inactivation kinetics of FMDV was conducted as follows: the log_10_ infectivity of the timed samples was plotted against time, and extrapolation was used to ensure that there would be <1 infectious particle per 10^4^ L of liquid preparation at the end of this inactivation period. For FMDV harvested from the shaking flask, 100 mL of FMDV supernatant was inactivated by adding various BEI concentrations (0.5–3.0 mM). The initial viral titer before BEI inactivation was 8.4 log TCID_50_/mL. The FMDV supernatant was then incubated in a shaking incubator at 75 rpm at 26 °C and 37 °C for 24 h. Next, 12 mL samples were collected at hourly intervals up to 6 and 24 h after BEI treatment. The residual BEI was neutralized with a 10% volume of 1 M sodium thiosulfate (Daejung Chemicals, Siheung-si, Korea) to a final concentration of 2%.

### 2.7. Animal Experiment

The purified Asia1 Shamir-R antigen (15 μg per dose) derived from a 2 L bioreactor was mixed with 1% saponin (Sigma-Aldrich) and 10% aluminum hydroxide gel (General Chemical, NJ, USA) to prepare a monovalent vaccine. The ISA 206 VG adjuvant (Seppic, Paris, France), pre-warmed at 30 °C, was then added at a ratio of 1:1, resulting in a 2 mL/dose of the experimental vaccine. The mixtures were incubated at 20 °C for 1 h in a water bath without light exposure and stored at 4 °C until use. Two-month-old pigs (*n* = 5) that had not been previously vaccinated against FMD were immunized intramuscularly twice at a 4-week interval with the Asia1 Shamir-R vaccine. The control group consisted of three unvaccinated pigs. Blood samples (10 mL) were collected at 0, 14, 21, 28, 35, 42, 49, and 56 d post vaccination (dpv) with a syringe and dispensed into heparinized tubes and centrifuged to recover sera.

Animal experiments in this study were approved by the Institutional Animal Care and Use Committee (IACUC) of the Animal and Plant Quarantine Agency (IACUC No. 2023-761).

### 2.8. Virus Neutralization Test

The virus neutralization (VN) test was performed as described in the WOAH terrestrial manual [13]. Sera were inactivated at 56 °C for 30 min before testing. Starting from a 1/8 dilution, sera were diluted in a two-fold dilution series across the plate, using two rows of wells per serum and a volume of 50 μL. Then, 50 μL of two-fold serially diluted sera were mixed with 50 μL of Asia1 Samir or Asia1/MOG/05-R containing 100 TCID_50_. After incubation at 37 °C for 1 h, 50 μL of LFBK cells (0.5 × 10^6^ cells/mL) was added to each well. The plates were sealed and incubated at 37 °C with 5% CO_2_ for 2–3 d. The VN titer was calculated as the reciprocal of the maximum dilution of serum that neutralized 100 TCID_50_ of FMDV and expressed as a log_10_ value.

### 2.9. Statistical Analysis

Each experiment was conducted in triplicate, and the means and standard deviations of all values are presented. Statistical data were analyzed using GraphPad Prism version 9 (GraphPad Software, La Jolla, CA, USA) for visual representation. Statistical significance was assessed using two-way ANOVA.

## 3. Results

### 3.1. Optimization of Conditions for Asia1 Shamir-R Proliferation

**Figure 1:**
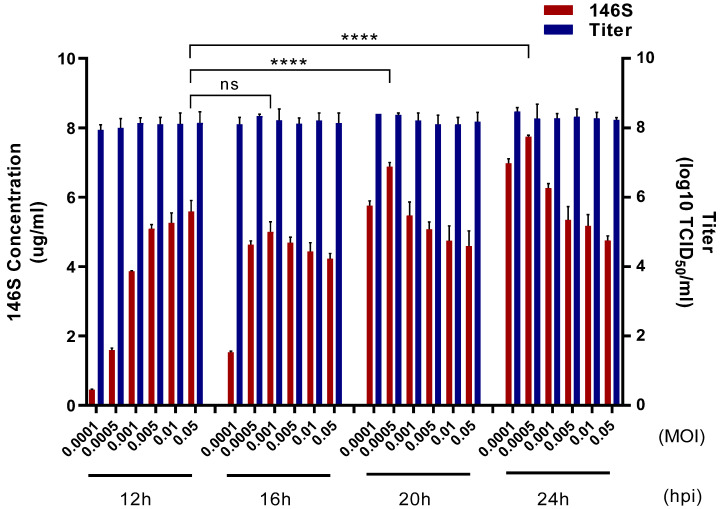
Optimization of the conditions for antigen production on a flask scale. The antigen yield and viral titer were determined according to the virus infection time and concentration. Blue bars indicate the virial titer. Red bars indicate the antigen yield. The results are presented as the mean ± standard deviation. Statistical analysis was conducted using an unpaired *t*-test (ns, not significant, **** *p* < 0.0001).

Asia1 Shamir-R was inoculated at different viral concentrations and recovered at different viral infection times to determine the antigen yield and virus titer to establish the optimal conditions for producing the Asia1 Shamir-R antigen (Figure 1). Under all conditions, the viral titer was >10^8^ TCID_50_/mL. The amount of antigen increased with increasing virus concentration only when the virus was infected for 12 h. In samples infected for 16 h or more, lower virus concentrations resulted in higher amounts of antigen, except at 0.0001 MOI. The optimal condition was 24 h of virus infection at a viral concentration of 0.0005 MOI, which yielded 7.7 μg/mL of antigen.

### 3.2. Comparison of Antigen Yield According to Production Size

**Figure 2:**
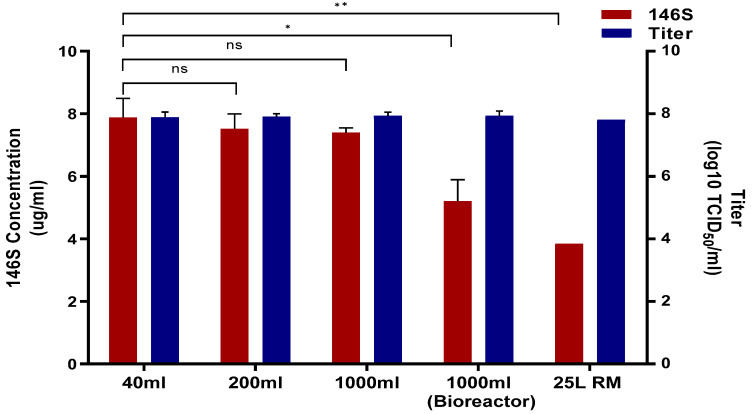
Comparison of antigen yield according to production size. The above conditions were applied to produce the Asia1 Shamir-R antigen from the flask scale to a 2 L bioreactor and a wave bioreactor with an RM bag. Red bars indicate the antigen yield. Blue bars indicate the virial titer. The results are presented as the mean ± standard deviation. Statistical analysis was conducted using an unpaired *t*-test (ns, not significant, * *p* < 0.05, ** *p* < 0.01).

The above conditions were applied to produce the Asia1 Shamir-R antigen from a flask scale, a 2 L bioreactor, and a wave bioreactor with an RM bag (Figure 2). Scaling up from the flask in five-fold increments, the virus titers at 40, 200, and 1000 mL were all 7.8 log TCID_50_/ mL, and the amounts of antigens were 7.8, 7.5, and 7.4 μg/mL, respectively. Using a 2 L bioreactor to grow Asia1 Shamir-R in a 1 L volume, the virus titer was 7.8 log TCID_50_/ mL and the amount of antigen was 5.2 μg/mL. When Asia1 Shamir-R was grown in a 25 L wave bioreactor with a disposable RM bag, the virus titer was 8.6 log TCID_50_/mL, and the amount of antigen was 3.8 μg/mL.

### 3.3. Inactivation Kinetics of Asia1 Shamir-R

**Figure 3:**
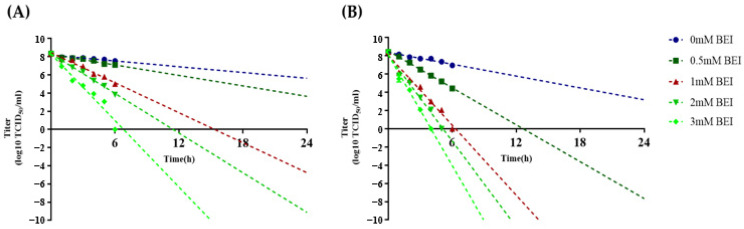
Inactivation kinetics of the Asia1 Shamir-R. The supernatant obtained after the Asia1 Shamir-R was inoculated in the BHK-21 suspension cells was inactivated by each binary ethyleneimine concentration, with samples taken hourly up to 6 and 24 h at 26 °C (**A**) and at 37 °C (**B**). The extrapolation of the individual graphs was drawn as a linear line for the analysis of FMDV inactivation kinetics.

During viral inactivation, samples were collected hourly up to 6 and 24 h to monitor the rate and linearity of the inactivation process. The viral titers before inactivation were 8.4 log TCID_50_/mL for Asia1 Shamir-R (Figure 3). The viral titer decreased faster at 37 °C than at 26 °C when treated with the same BEI concentration. In addition, the viral titer decreased rapidly as the BEI concentration increased, regardless of temperature. The criterion for FMDV inactivation is a decrease in the viral titer to −7 log TCID_50_/mL within 24 h of BEI treatment [13]. When virus inactivation was performed at 26 °C, treatment with 2 mM BEI exhibited a decrease in viral titer to −7 log TCID_50_/mL within 24 h (Figure 3A). When inactivation of FMDV was performed at 37 °C, the virus demonstrated a decrease in viral titer to −7 log TCID_50_/mL within 24 h of inactivation with 0.5 mM BEI (Figure 3B). We also investigated the changes in vaccine antigen yield depending on the inactivation conditions (Table 1). There was approximately 24% and 22% antigen loss from the initial antigen yield of 8.3 µg/mL at 26 °C and 37 °C, respectively. However, this loss was not due to BEI treatment because the samples without BEI treatment also demonstrated equivalent amounts of antigen loss under the same conditions.

### 3.4. Immunogenicity of the Asia1 Shamir-R Antigen in Pigs

**Figure 4:**
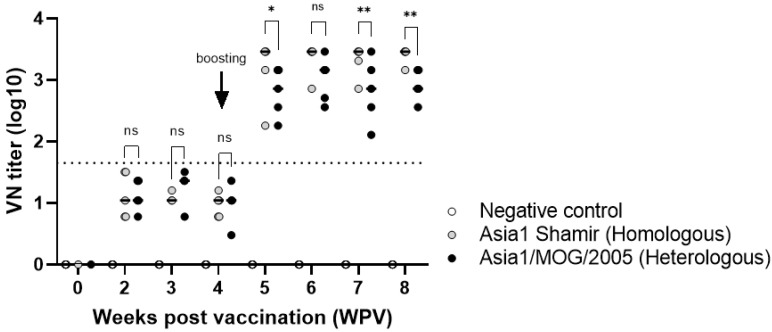
Virus neutralization titers post immunization with the Asia1 Shamir-R vaccine. Virus neutralization tests against Asia1 Shamir and Asia1/MOG/05-R viruses were performed using sera collected weekly from pigs immunized twice with the Asia1 Shamir vaccine at 4-week intervals. Data are presented as the mean ± standard deviation. The dotted line indicates the 1.65 log VN titer. Gray bars indicate the VN titer against Asia1 Shamir. Black bars indicate the VN titer against Asia1/MOG/05-R. Statistical analysis was conducted using an unpaired *t*-test (ns, not significant, * *p* < 0.05, ** *p* < 0.01).

Pigs were immunized with Asia1 Shamir-R antigens derived from a 2 L bioreactor, and the sera collected at weekly intervals were examined for the VN test. A single experimental vaccine dose resulted in VN titers of 1/45 or less against both Asia1/MOG/05-R and Asia1 Shamir (Figure 4). However, booster immunization of pigs with the vaccine resulted in VN titers of around 1/1000 against both Asia1/MOG/05-R and Asia1 Shamir.

## 4. Discussion

Since the massive FMD outbreak in 2010–2011, all FMD-susceptible livestock in the country have been immunized with FMD bivalent vaccines containing FMDV types O and A. However, in case of an outbreak of FMD type Asia1 that did not occur in South Korea, type Asia1vaccine has been stockpiled overseas in the form of an antigen bank. If a domestic FMD vaccine factory is built in the near future, it will be necessary to construct its own antigen bank. Therefore, the recombinant Asia1 vaccine strain, called Asia1 Shamir-R, was previously developed [8]. This study aimed to determine whether Asia1 Shamir-R can be commercially utilized for the FMD vaccine. First, we investigated the optimal conditions for flask-scale antigen production. Cellvento medium is the most favorable because it does not require medium exchange during the viral inoculation step and only requires 30% addition, which shortens the processing time and saves medium usage [14]. Antigens over 7 ug/mL were obtained during the flask stage using this medium, which is much higher than other reports that the antigens in the FMDV-infected supernatant were around 2 ug/mL [15,16]. Some cases have also been reported with antigens as low as 1 ug/ mL [17,18].

Although there was no difference in the virus titer as the virus culture size increased, the antigen yield decreased. The most important FMD vaccine component is the whole virus particle (146S) of the FMDV antigen. Therefore, other studies have shown that even if the FMDV titer is high, the protective efficacy of the FMD vaccine is significantly reduced if the number of 146S particles is low [19]. The reason for the decrease in antigen amount as the virus culture size increased may be the difference in the operation of each device; however, the exact cause is unknown. Similarly, the flask method has been reported to be more productive than bioreactors or wave bioreactors for influenza virus growth [20]. These results are not universal; in some cases, flasks and bioreactors are similar, and wave bioreactors are less productive [21]. Other studies have reported similar productivity in flask and wave bioreactors [22]. There are also reports that antigen yield can be increased by changing the detailed conditions, such as the rocking angle and rocking rate of the bag on the wave bioreactor [23]. In this study, only the optimal virus concentration and infection time selected at the flask scale were applied, and the optimal wave bioreactor conditions were not employed. This seemed to result in a relatively lower antigen yield in the wave bioreactor than in the flask.

The antigenic yield of around 4 ug/mL regardless of the culture method proves that the antigen productivity of Asia1 Shamir-R is superior to any other known foreign vaccine strain. Although a 2 L bioreactor was used in this study, the antigen yield of over 5 ug/mL might be 2–3 times higher than the antigen amount of foreign FMD vaccine strains. This indicates that Asia1 Shamir-R is a commercially valuable vaccine strain, as it lowers the cost of vaccine production. As other researchers have shown, the amount of antigen does not differ significantly even when the size of the bioreactor increases [24,25]. Therefore, a high antigen yield derived from a 2 L bioreactor using Asia1 Shamir-R can be reproduced in a vaccine factory using an industrial-scale bioreactor.

Because the FMD vaccine is manufactured through the process of virus inactivation, it is necessary to measure viral inactivation in the finished product. However, the FMD vaccine is produced by culturing FMDV in a large volume and working in a biosafety level 3 facility; therefore, another validation method for virus inactivation, called inactivation kinetics, should be performed during the course of antigen production [26].. We evaluated the BEI-dependent inactivation of Asia1 Shamir-R at 37 °C and 26 °C based on the reason demonstrated in the previous report [27]. We observed a rapid decrease in viral titer with increasing BEI concentration and faster inactivation at 37 °C compared to 26 °C that was the same as previous reports [17,28,29]. Antigen loss in Asia1 Shamir-R (Table 1) was not due to BEI treatment, because the samples without BEI treatment also demonstrated approximately the same amount of antigen loss under the same conditions. In contrast with the relative stability of Asia1 Shamir-R, a previous study reported that >60% of the antigen was lost after 24 h of treatment with 1 mM BEI at 37 °C [29]. In addition, the O1 Campos viral strain exhibited an antigen loss of >80% following incubation at 37 °C for 24 h even without BEI treatment [30].

As shown in Figure 4, the inactivated Asia1 Shamir-R antigen derived from a bioreactor instead of a flask was still immunogenic in pigs. The VN titer was less than 1/45 after a single dose but increased to approximately 1/1000 after a second dose, suggesting that double immunization with Asia1 Shamir-R in pigs can provide broad protection against various genotypes of type Asia1 viruses. It was previously reported that a vaccine strain in which VP1 of the recombinant Asia1/MOG/05 virus was replaced with VP1 of Asia1 Shamir virus was also effective against Asia1 Shamir virus when vaccinated twice in pigs [10]. The Asia1 Shamir vaccine has low antigenic similarity to Asia1/MOG/05 in cattle [31]. However, in the present study, sera from pigs vaccinated with Asia1 Shamir showed similar VN titers against Asia1 Shamir and Asia1/MOG/05 viruses. Another study reported that sera from pigs vaccinated with one or two commercial Asia1 Shamir vaccine doses exhibited similar VN titers against Asia1 Shamir and Asia1/MOG/05 [32]. Although there was not any difference in the amino acid sequences between Asia1/MOG/05-R viruses derived from a shaking flask and bioreactor, further study to evaluate protective efficacy by challenging the viruses in vaccinated pigs using the antigen derived from a bioreactor needs to be carried out in the future

Taken together, Asia1 Shamir-R could be used as a candidate vaccine strain for domestic antigen banks when domestic FMD vaccine production facilities are completed in the near future.

## Figures and Tables

**Table 1:** Vaccine antigen yield (µg/mL) in virus-infected supernatants after BEI treatment at 26 °C and 37 °C for 6 h and 24 h. Data are presented as the mean ± standard deviation.

BEI Concentration	26 °C			37 °C		
	0 h	6 h	24 h	0 h	6 h	24 h
0.0 mM BEI	8.3 ± 0.31	7.2 ± 0.15	6.2 ± 0.20	8.3 ± 0.31	7.0 ± 0.06	6.5 ± 0.25
0.5 mM BEI	8.3 ± 0.31	6.9 ± 0.32	5.9 ± 0.10	8.3 ± 0.31	6.9 ± 0.19	6.5 ± 0.51
1.0 mM BEI	8.3 ± 0.31	6.6 ± 0.21	6.1 ± 0.30	8.3 ± 0.31	6.6 ± 0.37	6.5 ± 0.21
2.0 mM BEI	8.3 ± 0.31	6.4 ± 0.32	6.3 ± 0.27	8.3 ± 0.31	6.4 ± 0.35	6.2 ± 0.25
3.0 mM BEI	8.3 ± 0.31	6.5 ± 0.10	6.3 ± 0.35	8.3 ± 0.31	6.4 ± 0.25	6.5 ± 0.12

## Data Availability

Data are retained within this article. The raw data are available from the corresponding author.
